# Supplementary material for: Altered microRNA Transcriptome in Cultured Human Liver Cells upon Infection with Ebola Virus
Source: Int J Mol Sci. 2021 Apr 6;22(7):3792. doi: 10.3390/ijms22073792 (PMC8038836; doi:10.3390/ijms22073792)
Supplement: Supplementary file 1 [file ijms-22-03792-s001.zip › Supplementary_File/C_ GO_Analysis_Results/16-30nt_go_Mayinga-96h-Huh7_vs_Control-96h-Huh7_up.mature_mirna_targets/GeneOntology_EnrichmentScore.pdf]

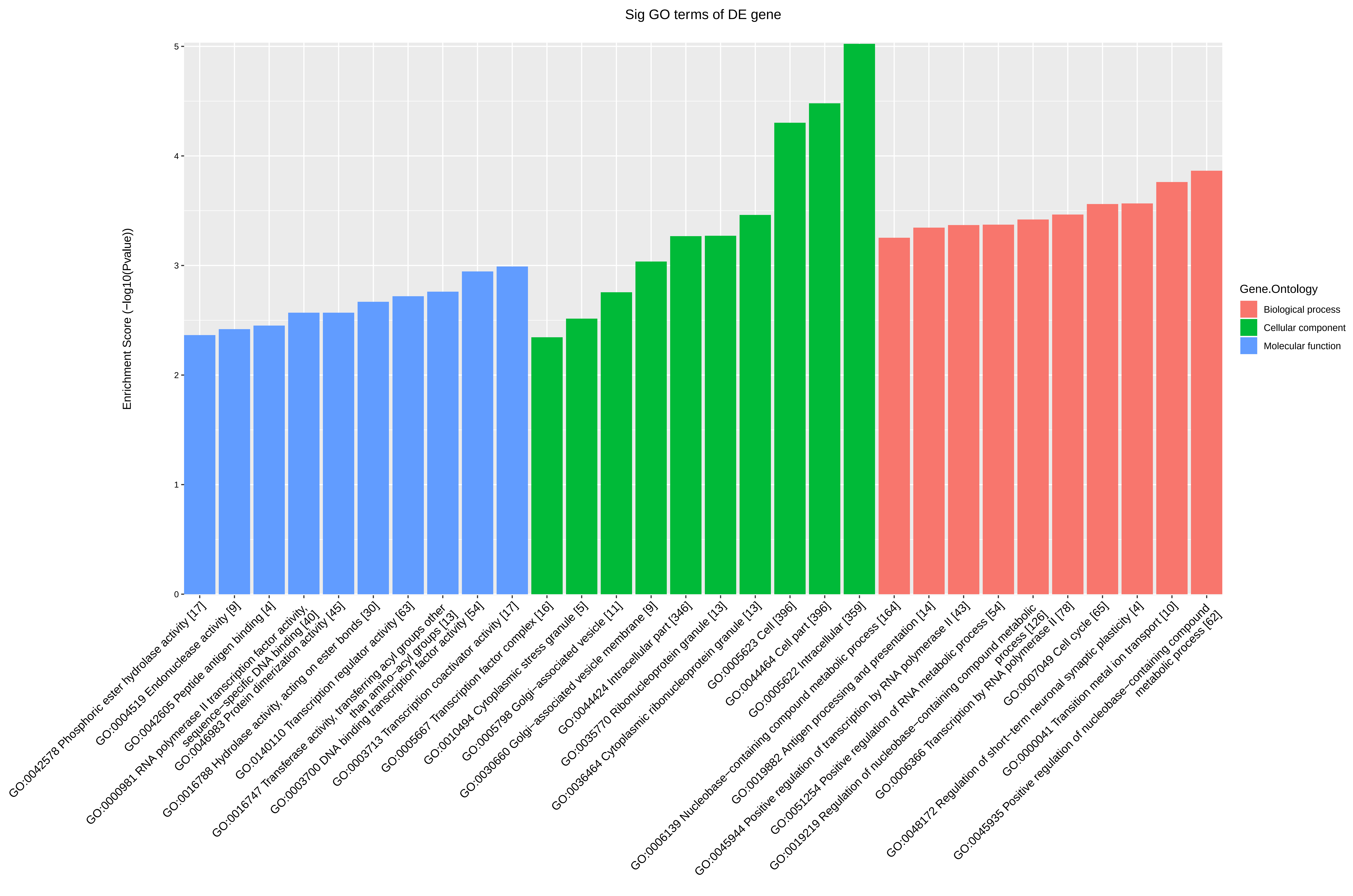

Sig GO terms of DE gene
Enrichment Score (−log10(Pvalue))
0
1
2
3
4
5
GO:0042578 Phosphoric ester hydrolase activity [17]
GO:0004519 Endonuclease activity [9]
GO:0042605 Peptide antigen binding [4]
GO:0000981 RNA polymerase II transcription factor activity, sequence−specific DNA binding [40]
GO:0046983 Protein dimerization activity [45]
GO:0016788 Hydrolase activity, acting on ester bonds [30]
GO:0140110 Transcription regulator activity [63]
GO:0016747 Transferase activity, transferring acyl groups other than amino−acyl groups [13]
GO:0003700 DNA binding transcription factor activity [54]
GO:0003713 Transcription coactivator activity [17]
GO:0005667 Transcription factor complex [16]
GO:0010494 Cytoplasmic stress granule [5]
GO:0005798 Golgi−associated vesicle [11]
GO:0030660 Golgi−associated vesicle membrane [9]
GO:0044424 Intracellular part [346]
GO:0035770 Ribonucleoprotein granule [13]
GO:0036464 Cytoplasmic ribonucleoprotein granule [13]
GO:0005623 Cell [396]
GO:0044464 Cell part [396]
GO:0005622 Intracellular [359]
GO:0006139 Nucleobase−containing compound metabolic process [164]
GO:0019882 Antigen processing and presentation [14]
GO:0045944 Positive regulation of transcription by RNA polymerase II [43]
GO:0051254 Positive regulation of RNA metabolic process [54]
GO:0019219 Regulation of nucleobase−containing compound metabolic process [126]
GO:0006366 Transcription by RNA polymerase II [78]
GO:0007049 Cell cycle [65]
GO:0048172 Regulation of short−term neuronal synaptic plasticity [4]
GO:0000041 Transition metal ion transport [10]
GO:0045935 Positive regulation of nucleobase−containing compound metabolic process [62]
Gene.Ontology
Biological process
Cellular component
Molecular function
